# Supplementary material for: Are carbon emissions trading and green financial instruments synergistic? -Comprehensive quantitative research based on content analysis
Source: PLoS One. 2024 Mar 7;19(3):e0298601. doi: 10.1371/journal.pone.0298601 (PMC10919601; doi:10.1371/journal.pone.0298601)
Supplement: S1 Data — (ZIP) [file pone.0298601.s001.zip › Data/地级市Z_F工作报告.zip!/║╙▒▒/└╚╖╗╩╨/└┤╫╘╣1⁄2╓┌║┼í╛┬φ┐╦╩2╛▌═°í┐_2010.docx]

各位代表：

现在，我代表廊坊市人民政府，向大会作政府工作报告，请予审议，并请市政协委员和列席会议的同志提出意见。

一、2009年工作回顾

刚刚过去的一年，是新世纪以来我市经济发展最为困难的一年。面对金融危机、甲流威胁的严峻形势和经济增长、国庆安保的艰巨任务，全市上下深入落实科学发展观，唱响“保当期增长、促科学发展”的主旋律，化危为机、攻坚克难，保持了全市经济社会平稳较快发展的良好势头。胡锦涛总书记亲临视察并对我市工作给予充分肯定。

（一）保增长任务圆满完成。全市经济在困难形势下跨越诸多障碍，呈现先期反弹、逐季回升、好于预期的可喜走势。全市地区生产总值完成1160亿元，同比增长10.8%；全部财政收入143.4亿元，增长17.4%，其中地方一般预算收入69.3亿元，增长19.5%；规模以上工业增加值392.4亿元，增长14.7%；城镇固定资产投资1172.5亿元，增长39%；社会消费品零售总额354.5亿元，增长18.1%；城镇居民人均可支配收入18333元，农民人均纯收入6834元，分别增长12.5%和11%。主要指标增速保持全省前列，规模以上工业增加值居第一位，地方一般预算收入居第二位，全部财政收入、城镇居民人均可支配收入和社会消费品零售总额均居第三位。全市用电量增速全省第一，贷款增幅、增量分居第一位和第三位。

（二）产业结构持续优化。编制实施服务业发展规划和工业倍增计划，以项目、产业、园区为抓手，同步推动经济增长和产业升级，金字塔型现代产业体系已具雏形。项目质量显著提高，实施投资千万元以上项目2264项，同比增加394项，其中亿元以上项目占30.6%，高新技术、装备制造、现代服务和现代农业项目占86%。重大项目建设进展加快，富士康产业基地一期投产，新奥薄膜太阳能电池投产并成功进入国际市场，华为产业基地投入运营效益良好，中兴产业基地、首钢装备制造业基地、廊坊科技谷、燕达健康城等项目建设进展顺利。产业升级加速推进，电子信息产业增加值增长53.1%，占到全省同行业的33.4%，装备制造、健康休闲、现代物流、金融服务等新兴产业蓬勃发展。园区承载力进一步提升，全市新增4个省级产业聚集区，5个园区实现扩区，廊坊开发区升级为国家级开发区。科技创新能力进一步增强，建成全国首家煤气化国家重点实验室、全省首家国家技术转移示范中心和专利技术展示交易中心，国家级家具质检中心落户香河。节能减排取得新进展，单位生产总值能耗完成省下达计划任务，二氧化硫排放削减5%，化学需氧量削减15.8%，在全省率先实现县县有污水处理厂。

（三）三年大变样扎实推进。把三年大变样作为加快城镇化进程的战略任务，打响拆迁整治、旧城改造、新城开发三大战役，全年完成基础设施投资158.8亿元，是我市城建史上投入最多的一年。启动7个城中村和光明片区、银河桥棚户区改造，完成拆迁225.7万平方米，腾出土地3853亩。实施了迎宾大道提升改造，完成新华路立面改造、和平路绿化提升等12项城市精品工程。市区新增树木500万株、绿地230万平方米，人均公共绿地面积达到12.7平方米。高速公路建设加速推进，市区南北交通和外围路网实现全面升级。县级三年大变样建设步伐加快，引进了上海绿地、深圳万科等一批战略投资者，积极推进城镇建设和村庄整理，城镇面貌明显改观。市级投融资平台建立，实现融资93.3亿元，各县（市）也都建立了相应公司，城建投融资实现历史性突破。

（四）农村经济稳步发展。全面落实强农惠农政策，发放各类支农补贴和家电、汽车、摩托车下乡补贴4.5亿元。粮食生产连续六年丰收，总产达到187.6万吨，增长5.5%。农业结构得到优化，新增造林30万亩，肉、蛋、奶、菜、果等主要农产品产量平稳增长，农业生态旅游持续升温。农业产业化水平继续提高，规模以上企业达到170家，带动农户50万户。农村改革稳步推进，县、乡、村三级土地承包经营权流转市场基本形成，完成土地流转45万亩。全民创业活力增强，新建创业辅导基地4家，完成创业培训18.3万人次，新增2个中国驰名商标、26个省著名商标，千人拥有民营企业数达到5.7家。新民居建设顺利推进，完成投资34.5亿元，80个省级示范村街建设别墅式、公寓式新民居985栋，农村生产生活环境不断改善。

（五）改革开放不断深化。国企改制工作进展顺利，蓟县冶炼厂完成产权出让，明珠集团和商贸集团完成清产核资。财税体制改革继续深化，政府机构改革有序推进。着眼深度整合京津资源，全力推动规划、产业、交通对接。与北京签署《城乡规划合作交流机制框架协议》。成功举办北京•廊坊周，签约项目投资额达到302.8亿元。京唐高铁在香河设站，京廊轨道交通L2、M6线对接工作积极推进。

（六）民生保障持续改善。扎实推进六方面18项民生实事，完成农村劳动力向非农产业转移14.8万人次，城镇登记失业率控制在2%以内。城镇低保对象全部免费参加城镇居民基本医疗保险，农村低保对象免费加入新农合，新农合参合率达到87.4%。城乡低保实现应保尽保，五保户集中供养率保持全国领先水平。投资3000万元，建成高标准的市级社会福利院。建设经济适用住房41.5万平方米，在全省率先全部解决城区低收入家庭和农村特困、灾贫户住房困难。住房公积金保障作用不断增强，累计归集37.6亿元。投资1.1亿元建成10座水厂，解决了21万人的饮水安全问题。

（七）社会建设全面加强。我市在全国114个城市公共文明暗访测评中，列地级市第三位。启动实施了中小学校舍安全工程，完成3所市区中小学扩建和34所农村中小学标准化建设。我市连续四次通过全国科技进步先进市考核，主要科技指标创历史最好水平。农村卫生“三基两化”建设在全省率先达标，甲流防控工作取得胜利。文化信息资源共享工程覆盖面扩大，成功举办廊坊书法晋京展，新编历史剧《吕端》获省戏剧节全部6项最高奖。市体育场竣工，我市运动员在十一届全运会首获金牌。社会治安和信访稳定工作切实加强，我市被评为全国社会治安综合治理工作优秀市。安全生产和食品药品安全得到保障，建成省级食品安全检测中心。“计生惠民行动”深入实施，国防动员和双拥共建深入推进。民族宗教、外事侨务、广播电视、物价、档案、人防、气象、妇女儿童、残疾人、地方志等各项工作均取得了新进展、新成绩。

各位代表，过去的一年，我们深入学习实践科学发展观，扎实推进干部作风建设年活动，进一步加强了政府自身建设。行政审批项目削减68.5%，收费项目削减72.4%，分别减至135项和113项，我市成为环渤海地区行政审批事项最少的城市之一。在全省率先实施行政审批全程电子监察，大力规范行政处罚自由裁量权，执法环境得到优化。通过市长专线为市民解决问题23060项，办结率99%。加大督查考核力度，强化审计监督，加强廉政建设和反腐败工作，行政效能和服务水平有了新提高。自觉接受人大法律监督和政协民主监督，办理人大代表建议、政协提案433件，认真听取各民主党派、人民团体和社会各界意见，依法行政能力进一步增强。

各位代表，回顾一年的工作，困难前所未有，成绩来之不易。这是省委、省政府和市委坚强领导的结果，是全市人民团结奋斗的结果。在此，我代表廊坊市人民政府，向辛勤工作在各条战线上的广大干部群众，向人大代表、政协委员，向各民主党派、工商联、人民团体和各界人士，向驻廊解放军、武警官兵、公安干警和中省直单位，向所有关心支持廊坊发展的海内外朋友们，表示崇高的敬意和衷心的感谢！

二、2010年形势和任务

今年是“十一五”规划的最后一年，也是我市在新的起点上谋划“十二五”发展、加快推进“两个率先”的关键之年。

总体看，全市经济社会发展面临难得机遇。宏观经济回升向好，整体发展环境好于去年；省委、省政府强力推进现代产业体系建设和城镇面貌三年大变样，为我市加快发展提供了有力支撑。特别是北京启动实施城南行动计划和通州新城建设，为廊坊带来了历史上从未有过的重大机遇。城南行动计划，将北京南部作为首都未来发展的重要空间和京津冀合作的门户通道，三年投入2900亿元，重点建设7条轨道线路、27条跨区通道和主干道，并围绕发展城市南中轴产业带、永定河水岸经济带和高端产业园区，构建“一轴一带多园区”的发展格局。通州新城建设，将按照世界一流标准启动建设北京的新城区，并在运河核心区建设北京新的CBD，有8条轨道线路接入通州新城。城南开发和新城建设的实施，进一步加速了北京的产业转移、消费外溢和市场扩张，城市带动力将直接辐射我市，带来三个方面的新机遇，一是推动对接京津迈向全面融入的新阶段，二是为建设环京津城镇群注入强大动力，三是为产业发展特别是高端服务业提供了重大契机。着眼三大机遇，立足多年努力打造的城市、产业和交通平台，廊坊已经具备了冲刺跨越、全面争先的基础和条件。

我们也清醒地看到，廊坊发展面临着严峻挑战。整体实力较弱、自主创新能力不强的历史问题尚未得到根本解决，一些新问题、新挑战又逐渐凸显。京津、京石、京承、京张等重大交通设施建设，使廊坊的区位优势相对弱化。国际金融危机的深层次影响依然存在，利用外部资源困难较多。面对深刻变化的新形势，部分干部的精神状态和工作作风不适应发展的需要，缺少应有的危机感和紧迫感。放眼日益激烈的区域竞争态势，标兵渐远，追兵迫近，我们面对的挑战和压力比以往任何时候都更为紧迫、更加突出。

天时、地利、人和交集的大形势，昭示着多年以来梦寐以求的大发展、大跨越时机已经来临。虽然我们在发展中会遇到诸多困难，但通过努力都是可以克服的。能不能抓住机遇、用好机遇才是关键所在。我们需要坚定信心、趋利避害，乘势而上、抢占先机，在新一轮竞争中实现冲刺跨越、全面争先。

今年政府工作的总体要求是：全面贯彻党的十七大和十七届三中、四中全会精神，深入落实科学发展观，以冲刺跨越、全面争先为主基调，着力壮大经济规模和提升总体实力，着力优化经济结构和转变发展方式，着力推进改革开放和自主创新，着力改善民生和促进社会和谐，加速构筑“京津冀电子信息走廊、环渤海休闲商务中心”主框架，确保以绿色人均收入为主导的发展指标赶上或超过环渤海经济圈平均水平，努力在科学发展、富民强市进程中迈出更加坚实的步伐。

今年经济社会发展的主要预期目标是：地区生产总值增长10%以上；全部财政收入和地方一般预算收入增长15%以上；规模以上工业增加值增长15%以上；全社会固定资产投资增长20%以上；城镇固定资产投资增长22%以上；城镇居民人均可支配收入、农民人均纯收入分别增长10%和8%以上；单位生产总值能耗、化学需氧量、二氧化硫排放量全面完成“十一五”规划目标。

围绕上述要求和目标，今年政府工作的首要任务是，既要保持经济平稳较快发展，又要在转变发展方式上取得突破。在坚持以往行之有效的工作思路基础上，紧紧抓住新的发展机遇，突出建设好“四大体系”：一是大力推动产业结构优化升级，加快构建现代产业体系；二是大力推进城镇面貌三年大变样和新农村建设，加快构建现代城镇体系；三是大力推进京津廊、城乡和市境交通一体化，加快构建现代交通体系；四是进一步为人民群众织密福利安全网，加快构建以解决各项民生困难为重点的社会性保障体系。

三、2010年重点工作

今年经济社会发展的任务十分繁重，必须紧紧围绕全年目标任务，立足全局谋长远，集中精力干大事，抓住关键求突破。重点做好六个方面的工作。

（一）以更大的力度增投入、上项目，保持经济平稳较快增长。

围绕“四大体系”建设，谋划实施重大项目，力求取得更大突破。全年实施建设投资千万元以上项目2000项，其中亿元以上项目550项。

一是推进重大项目建设。集中力量抓好98个投资10亿元以上的重大项目。对首钢装备制造业基地、中兴产业基地、燕郊空港物流园、亚太家具材料城等22个在建项目，加强协调调度，强力组织推进，力争如期建成投产；对霸州吉利汽车产业基地、国际信息云聚核港、香河国际农产品交易中心等30个计划新开工项目，千方百计落实开工条件，争取尽早开工建设；对广阳海尔物流中心、固安天赐温泉城、京东数字文化创意产业园等46个前期谋划项目，积极协调推进，确保取得实质性进展。通过实施一批产业关联度高、带动力强的新项目，为经济平稳较快增长提供支撑，为廊坊长远发展积蓄后劲。

二是积极谋划和争取新项目。围绕国家扩大内需的后续政策，谋划和储备一批符合国家产业政策和投资方向的重大项目，加强沟通，加紧申报，争列国家和省“十二五”盘子。按照省申列重点项目的要求，充实项目库，完善前期手续，确保省重点项目数量位居全省前列。增强项目引进的针对性和实效性，围绕电子信息、新能源、装备制造等主导产业，瞄准世界500强、中央大企业、北京大集团，力争引进1-2家龙头型、基地型项目。

三是健全项目推进机制。实行市级领导分包重大项目责任制，确保责任落实到位。实行产业招商责任制，按产业分类谋划引进项目，把任务落实到各县（市、区）、重点园区和相关部门。进一步完善项目考核机制，严格执行项目考核办法，切实兑现奖惩措施。

四是提高园区建设水平。启动实施《廊坊市园区空间布局总体规划》，全面提升17个重点园区规划建设水平。廊坊开发区和燕郊开发区以增强活力、壮大实力为目标，推行管办分离、政企分开、市场运作，在机制再造和体制创新上迈出新步伐，积极推进燕郊开发区升级为国家级高新区。对新获批的省级园区和产业聚集区，围绕“规划、机制、人才”三要素，按照“五有”目标要求，加强基础设施建设，完善各项服务功能，增强对重大项目的承载能力。各县（市、区）加强资源整合，集中力量打造样板园区，培育新的县域经济增长极。继续做好园区扩区工作，抓住省第二批园区扩区的契机，力争大厂、永清、文安、大城四个工业园区实现扩区。进一步加强园区考核，实行分类指导，定期公布园区主要指标排位，年终兑现奖惩。

五是大力推动全民创业。实施中小企业成长工程，为全民创业提供土地、资金、技术等要素服务，打造服务全承办、审批零收费、建设零干扰、发展无障碍的创业环境。加快创业辅导基地和创业园区建设，全市新建标准化创业辅导基地4个，建成乡镇产业园20个、村街创业园50个。加强融资服务，壮大担保机构和小额贷款公司规模，筹建1-2家村镇银行；落实好上市融资的扶持政策，推进梅花、华夏等企业上市工作。开展多种形式的创业培训，完成培训18万人次。全市新增注册民营企业2000家以上，千人拥有民营企业数量保持全省领先水平。

（二）推进产业结构优化升级，加快构建现代产业体系。

围绕构建金字塔型现代产业体系，着力优化提升传统产业，培育壮大新兴产业，推动企业创新和产业升级迈出实质性步伐。

一是推进信息产业基地建设。围绕构建“京津冀电子信息走廊”，发挥国家级信息产业基地的品牌优势，不断提升产业规模和聚集度。以富士康、华为等大项目为龙头，以重点园区为载体，狠抓上下游配套和产业链整理延伸，加快建设永定信息产业基地。突出做好富士康文章，多措并举，高效服务，确保富士康增资尽快到位，积极促成手机生产基地北迁廊坊，千方百计满足富士康用工需求，力争全年产值超过100亿元。加快富士康生产生活配套建设，台资工业园标准厂房今年竣工9万平方米，聚集配套厂商20家以上；启动总投资11亿元的配套生活区项目，满足富士康及其配套厂商的生活需求。建设好深港工业园，争取香港高迪数码等一批重大项目尽快落地开工。全年电子信息产业主营业务收入达到225亿元。

二是壮大现代服务业。围绕建设环京津休闲商务产业带，推进燕达健康城、汇福健康城和成功大广场、霸州温泉公园等重大项目建设。着力发展现代物流业，加快建设胜芳国际物流中心、京东物流中心等重点项目，引进、培育大型物流企业，完善物流配送体系。提升会展旅游业，整合提升会展旅游设施，全力办好5•18、9•26、城博会、APEC企业家峰会、国际电梯展等展会，推进旅游重点项目建设。大力发展金融业，积极实施“引金入廊”工程，加快发展各类投资基金，建设面向北京的金融后台服务基地。积极培育文化产业，编制好全市文化产业发展规划，加快万庄文化生态旅游产业园规划建设，扶持推动2-3个市场前景好、发展潜力大的文化产业项目。积极发展总部经济，发挥近邻京津、宜居宜业、服务高效的优势，制定和完善优惠政策，推动总部基地建设。

三是发展新型农业。按照城郊—都市型农业的发展目标，在稳定粮食生产的基础上，重点发展健康畜牧养殖、无公害蔬菜、优质粮油、生态林果花木四大主导产业。大力发展设施型农业，新增节水灌溉面积10万亩。培育扶持农产品加工龙头企业，新上一批大型农业项目，建设农产品加工园区，壮大产业基地规模，形成区域性特色产业集群。积极发展新型农业业态，探索庄园型、工厂型、公司型等农业先进模式，在发展有机农业、创意农业、订单农业等方面取得新突破。努力推进农业科技创新，加快新品种、新技术、新设备的研发和成果转化，增强农业综合竞争力。

四是培育新兴产业。抓住国家支持战略性新兴产业发展的机遇，围绕新能源、新材料、生物医药等重点产业，加快新奥煤基清洁能源、福尔制药、梅花生物制药、京冶科技、盛唐高分子材料等重点项目建设，积极谋划引进一批新的龙头项目，壮大产业规模，完善产业链条，培育产业集群。借鉴金隅工业园建设模式，实行以商招商，用龙头聚项目，建设新兴产业聚集区，打造新的竞争优势和经济增长点。

五是大力发展创新型经济。发展创新型经济是加快发展方式转变、实现又好又快发展的必然选择。着眼提升全市经济长远竞争力，启动实施创新廊坊计划，充分发挥政府引导作用，创新发展理念，集聚创新资源，汇聚创新人才，营造创新环境，培育以技术论英雄、以创新为荣耀的社会氛围，形成政府鼓励创新、社会尊重创新、企业追求创新、人人勤于创新的生动局面。

建设创新创业和技术研发“两大平台”。强化清华科技园和廊坊科技谷创新创业综合服务功能，全市新建企业孵化器5家，省级以上企业孵化器达到10家，打造环京津科技创新和项目孵化基地。支持新奥光伏、迪诺普、晶日金刚石等20家企业建立技术研发中心，支持新奥煤气化国家级重点实验室、省燃料电池实验室、省矿井灾害防治实验室等10家省级以上实验室建设。

强化核心技术和高层人才“两个支撑”。围绕电子信息、新能源、装备制造等主导产业加强技术攻关，推动煤基清洁能源技术等项目申报国家科技支撑计划，力争取得核心技术100项，促进“廊坊制造”向“廊坊创造”转变。实施培养和引进高层次人才的鼓励政策，逐步建立开放、流动、协作、竞争的人才机制，全面推行技术入股和科技人员持股经营制度，试行股权、期权等产权激励制度，最大限度地激发科技人员的首创精神。

开展政研企对接和企业对标 “两项活动”。组织科研院所、重点院校与企业进行有效对接，为产品技术升级和科研成果转化搭建平台。引导企业对标先进，开展“学新奥、练内功”活动，加强管理创新和技术创新，增强企业核心竞争力。三到五年内，培育100家在全国技术领先、竞争力强的创新型企业。

六是打好节能减排攻坚战。按照循环经济发展理念，加大节能减排工作力度。重点抓好前进钢铁、梅花味精、三河电厂、霸州新利4家省“双百”企业节能工程；对42家用能大户企业加强监督检查，深入实施综合节能改造；推进文安、霸州“双三十”节能减排示范工程。落实节能减排问责制，严格控制单位生产总值能耗，加快淘汰落后产能，切实防止高耗能、高排放项目上马。大力推广使用高效节能技术和产品，推进太阳能电池等新型清洁能源技术在我市推广应用。

（三）以深入推进三年大变样为抓手，着力加快城镇化进程。

今年是三年大变样的决战之年。按照服务发展、适宜人居、优美环境的要求，重点实施城市发展、功能完善和民生保障10大项、100个子项工程，总投资800亿元，其中政府主导投资130亿元，完成拆迁200万平方米以上。

一是在规划水平上有新提升。按照“生态、智能、休闲、商务”的功能定位，大力提升城市规划的特色和水平。聘请美国霍克公司以城乡一体化发展为核心，编制生态智能城市规划。做好奥林匹克环城公园规划和休闲中心概念性规划，全面整合环城林带、水系等景观资源，完成京沪高铁两侧用地一体化规划，努力形成“长廊呈秀、环绿争春、龙凤来朝”的独有城市风貌。

二是在城市功能上有新跨越。加大中心区改造提升力度，重点建设投资50亿元的广阳CBD、投资30亿元的中心商业区、投资6.3亿元的龙河水系景观带、投资5亿元的奥林匹克环城公园、投资5亿元的植物园项目，以地标性精品工程提升城市品位。着力推进南城开发，重点实施光明商务区、龙河中心区、银河新区和高铁广场商贸区等项目。加强市区交通建设和管理，打通市区断头路，新建5条次干道，建立多部门协调联动的交管机制，有效缓解堵车问题。加大迎宾线改造提升力度，高标准、大手笔提升光明道两侧景观带。加快万庄新城建设，以文化休闲和科技创新产业为导向，以重大项目为支撑，对万庄新城进行统一规划，积极推进文化产业园、悦榕庄酒店等项目建设。着力破解水资源制约，建设南水北调天津干线引水工程，确保廊坊干渠开工建设，加快推进广阳水库立项工作，力争实现引黄调水常态化。

三是在城镇体系建设上有新进展。加快建设固安、永清和主城区直通高速公路，构筑大城区组团空间格局，积极打造半小时交通圈。在县县通高速的基础上，把高速交通向园区延伸，努力实现重点园区通高速公路。抓住对接京津的新机遇，编制和实施环京津城镇群发展规划，选取一批具有良好区位优势和产业基础的重点城镇，结合村庄整理和新民居建设，建设一批高标准的小城市。加快人口向中心城市和重点城镇聚集，稳妥推进本地人口农转非，积极吸引京津高端人群，迅速提高城镇人口比重。通过三至五年的努力，形成以中心城市、县城、重点镇为框架，一体两翼、功能互补的城镇体系。

四是在北三县经济一体化上有新突破。加强北三县经济一体化规划，在重大基础设施、产业协调、城镇布局、共有河流和国道整治上，进一步做好规划和整合。研究出台税收分成的利益共享机制，促进项目统一摆放、产业统一布局。把燕郊新城作为环北京城镇群的重要节点，尽快编制新城规划，加快开发建设步伐。

（四）统筹城乡发展，切实做好“三农”工作。

以胡锦涛总书记视察我市新农村建设为动力，认真落实中央一号文件精神，加大统筹城乡发展力度，强化农业农村发展基础，提高农民收入，推动新农村建设迈上新台阶。

一是加强农业农村基础设施建设。完善农田水利设施，补打机井1000眼，维修改建闸桥、泵站11座，积极实施永定河、潮白河、子牙河险工加固等7项应急度汛工程，加强农田防护林网建设，提高农业综合生产能力和抗灾减灾能力。推广沼气、秸秆综合利用等可再生能源技术，加快建设大型沼气综合利用工程。加强农村公路建设，投资1.5亿元，新建改建农村公路300公里。大力实施通道绿化工程，打造精品绿廊。

二是持续推进农业产业化。以项目和品牌建设为重点，强龙头，兴园区，壮基地，全力提升农业产业化经营水平。切实抓好10个市级产业化重点项目，争取有1-2个列入省十大重点项目，扶持100家农产品加工龙头企业。重点建设20个种养基地、6个市级农产品加工园区和2个省级农产品加工基地县，扶持发展50个专业村。培育市级十大农产品品牌，争创国家级和省级名牌产品。着力提高农业生产经营组织化程度，大力发展农民专业合作组织，为农民提供便捷高效、质优价廉的专业服务。农业产业化经营率达到64%，带动农户51万户，农户参与度达到63%。

三是多渠道增加农民收入。认真落实农资、良种、农机具等各项补贴政策，及时拨付兑现家电下乡、汽车摩托车下乡等补助资金，稳定政策增收。挖掘农业内部增收潜力，大力发展农村二三产业，鼓励农民从事农产品加工和农村服务业，扩大产业增收。引导农村富余劳动力进城务工，全年转移13.8万人次，增加农民工资性收入。加大农村劳动力培训力度，完善技能式、创业式、委托式、订单式培训模式，完成一村两员、实用技术、科技示范户等培训30万人次以上。

四是深化农村各项改革。坚持最严格的耕地保护制度，加大土地执法力度。进一步完善农村土地流转市场，引导农民依法流转土地，流转率达到10%以上。全面深化集体林权制度改革，建立健全林业支持保护、林木采伐、林权流转、林业社会化服务体系等各项制度。普遍建立农村财富积累机制，规范村级集体资产财务管理，稳步开展农村集体产权制度改革。今年在全市70%的村街建立财富积累机制，明年基本实现村级全覆盖。

五是强力推进新民居建设。把新民居建设作为加速城镇化、推进新农村建设的突破口，坚持规划先行、产业带动、政策引导、因地制宜，以新农村先行示范区为牵引，以组团建设为基本导向，着力破解资金短缺、土地流转和市场对接等关键性难题，大规模推进新民居建设。今年重点抓好150个省级示范村工程，三年内使15%的村街达到省级示范标准。坚持把新民居建设与新产业、新农民、新体制、新文化统筹谋划，协调推动，努力实现新农村建设高水平、跨越式发展。

（五）全面深化对接京津，拓展对外开放深度和广度。

抓住北京城南开发和新城建设等新机遇，加快对接京津、同城发展步伐。积极搭建开放平台，加大招商引资力度，全面提高开放型经济水平。

一是深化规划对接。以京津为坐标系，对产业、城建、交通等各个方面进行统筹规划，确保规划上与京津统一协调，在发展上与京津互补共赢。密切与京津规划部门的沟通联系，加强与京津两市“十二五”规划的衔接，争取两市规划中更多体现京津廊合作意向，提高规划对接的主动性、超前性和实效性。高度关注北京新机场建设，编制好《临空产业区战略规划》，开展区域内产业、交通和空间布局研究。

二是加强产业对接。立足我市产业基础，重点抓好环京津高新技术产业带建设，实现高端产业配套协作，优势产业协调互补。谋划建设产业转移基地，建设承接京津产业转移的功能区、聚集区，探索项目转移、园区共建、互利合作的新机制和新途径，吸引京津高科技、高成长性企业聚集廊坊。加强旅游交流合作，扩大京津冀旅游“一卡通”发行，增加廊坊景区，扩大知名度。

三是加快交通对接。着眼构筑现代交通体系，完善路网结构，提升公路等级，打通道路接口，打造无障碍交通圈。加快轨道交通对接，全力推进京山铁路客运改造和京廊轨道交通对接等重大项目，抓好津保高速铁路霸州站点建设。加快高速公路对接，实施廊沧、京台、密涿支线、密涿（廊坊至北三县段）等高速公路建设。加快干线公路对接，推进采留线、104线改造、平香线南延、津保南线改造等项目前期工作，争取早日开工。加快京廊公交对接，开通廊坊市区至首都国际机场客运专线，把北京城市公交引入永清。

四是加大招商和城市营销力度。大力开展组团招商、以商招商、代理招商，全方位对接京津及国内外大企业、大财团、大机构，力争在重大项目引进上实现新突破。按照《廊坊市城市营销策划方案》，以更宽的视野、更大的力度开展城市营销，重点组织好北京•廊坊周、韩国•廊坊周等推介活动，打响城市、园区和产业品牌，提高廊坊的知名度、美誉度，增强招商引资的优势和吸附力。

（六）着力保障和改善民生，不断加强社会建设。

坚持把增进群众福祉放在政府工作的首位，着力推进以民生为重点的社会建设，全面提升人民群众幸福指数和社会文明程度。

一是着力改善民生。突出抓好就业、就医、就学、住房、养老五大惠民体系，实施危房帮建、扶残助残、饮水安全、校舍安全、医疗设施建设五项利民工程。

就业保障体系。继续开展“就业援助月”、“民营企业招聘周”等服务活动，引导企业吸纳就业，鼓励自谋职业、自主创业，全市城镇新增就业4.25万人，城镇登记失业率控制在2.5%以内。

医疗保障体系。继续提高农村卫生“三基两化”水平，社区卫生服务机构街道覆盖率达到100%，新农合参合率达到90%以上。继续资助农村低保户参加新农合，城镇低保对象免费参加新城合，实现城乡低保对象医保全覆盖。

住房保障体系。加大保障性住房供给，让困难群众住有所居，中低收入家庭住房条件不断改善。全年建设经济适用住房15万平方米，筹集实物廉租房1750套，新增归集住房公积金10亿元，发放住房公积金贷款7亿元。

养老保险体系。巩固养老保险省级统筹体制，积极推进事业单位养老保险制度改革。抓好新型农村社会养老保险试点，鼓励有条件的县（市、区）自行开展新农保，力争在全市范围全面启动，努力实现养老保险体系的全覆盖。

就学保障体系。完善孤残儿童救助机制，加快特殊教育学校建设。落实义务教育和中等职业教育贫困学生救助政策，开展多种助学活动，设立贫困大学生助学基金，不让一个学生因贫失学。努力提高人均受教育年限，高中阶段教育毛入学率达到88%以上。

危房帮建工程。汛期以前完成1731户农村困难群众危房帮建，提前一年完成三年目标任务。

饮水安全工程。投资1.5亿元，新建13座水厂，解决农村30万人的饮水安全问题。

扶残助残工程。把残疾人扶贫纳入政府扶贫计划，每年扶助6000名贫困残疾人，免费为3000名残疾人进行职业教育培训，安排1000名残疾人实现就业。

校舍安全工程。投资17.3亿元，启动638所中小学校舍修建工作，年内完成工程量的60%。

医疗设施建设工程。投资1亿元，建设市中医院综合病房楼，筹建廊坊市精神病医院；争取中央专项资金，支持3所县级医院建设。

二是加快发展各项社会事业。继续争创全国文明城市，深入开展群众性精神文明创建活动，进一步提高市民文明素质和城市文明程度。推进可持续发展实验区建设，做好中期验收准备。推进义务教育高水平均衡发展，提升普通高中教育质量，加快职业教育资源整合，努力促成人大附中分校落户。全力承办好十三届省运会。深入实施文化信息资源共享工程，加快完成村街基层服务点建设。落实人口和计划生育各项政策，稳定低生育水平，人口出生率控制在14.48‰以内。继续推进国企改革，实现经营性国有资产监管全覆盖。扎实推进医疗卫生体制改革，加快推进覆盖城乡居民的公共卫生服务体系、医疗服务体系和药品供应保障体系建设。总结运用甲流防控经验，建立应对突发公共卫生事件的长效机制。

三是维护安全稳定。完善领导大接访制度，建立健全矛盾大调解机制，抓好联合接访中心和巡特警队伍建设，强化社会治安综合治理，增强保障公共安全和处置突发事件的能力。进一步健全安全生产长效机制，强化执法监督，深化专项治理，完善应急救援体系。理顺食品药品监管体制，加大监管力度，坚决杜绝重大食品药品安全事故的发生。

四、切实加强政府自身建设

置身冲刺跨越、全面争先的关键时期，面对推动经济社会又好又快发展的艰巨任务，必须强化政府自身建设，全面提高组织推动科学发展的能力。

第一，进一步提高抢抓机遇的能力。树立强烈的竞争意识、拼抢意识，以更高的目标要求，自我加压谋发展，心无旁骛干事业，巧借天时，放大地利，凝聚人和，把廊坊的优势点变成新的增长点，把难得的机遇变成发展的动力。

第二，进一步提高创新发展的能力。跨越争先，贵在创新，重在落实。坚持把创新精神贯穿到政府工作全过程，搭建创新平台，建立创新机制，进一步激发创新创业创优的澎湃活力，发展创新型经济，建设创新型城市。坚持实干兴市，认准的事情扭住不放，决定的工作一抓到底，制定的目标必保完成，做到纵比有突破、横比有进位、结果有实效。

第三，进一步提高勤政善政的能力。大兴学习研究之风，提高科学判断形势、驾驭市场经济和应对复杂局面的能力。加强行政效能建设，全面实施行政审批电子监察，稳妥推进机构改革，形成权责一致、分工合理、决策科学、执行顺畅、监督有力的行政管理体制。推进信用体系建设，大力构建以政府、企业和重点人群为主要内容的社会信用监管平台，充分发挥信用产品在招投标等经济活动中的作用。集中各方面智慧，组织编制“十二五”规划，谋划好廊坊科学发展的新蓝图。

第四，进一步提高依法行政的能力。自觉接受人大和政协监督，广泛听取各界意见和建议，切实保障人民群众的知情权、参与权、表达权和监督权。建立和完善重大问题集体决策、专家咨询、社会公示和听证制度，对事关全局和人民群众切身利益的决策事项，广开言路，广纳民意。规范行政执法行为，做到严格执法、公正执法、文明执法，确保一切工作都在法律的框架内进行。

第五，进一步提高廉洁持政的能力。认真落实党风廉政建设责任制，构建重大项目、重大工程保廉体系，从源头上预防和治理腐败，坚决纠正损害群众利益、影响经济社会发展的不正之风。加强公务员管理和监督，完善考核体系和奖惩机制，建设一支廉洁高效的公务员队伍。

各位代表，一个振兴廊坊的重大机遇就在我们面前。历史机遇不容错过。让我们以胡锦涛总书记视察廊坊为动力，在省委、省政府和市委的坚强领导下，树立舍我其谁、勇于担当的责任感和使命感，以只争朝夕的精神、放马一搏的勇气，团结一心，真抓实干，共同创造廊坊更加美好的明天！
